# Greater neural pattern similarity to the native language is associated with better novel word learning

**DOI:** 10.3389/fpsyg.2024.1456373

**Published:** 2024-12-04

**Authors:** Yuan Feng, Aqian Li, Jing Qu, Huiling Li, Xiaoyu Liu, Jingxian Zhang, Jiayi Yang, Leilei Mei

**Affiliations:** ^1^Philosophy and Social Science Laboratory of Reading and Development in Children and Adolescents (South China Normal University), Ministry of Education, Guangzhou, China; ^2^Center for Studies of Psychological Application, South China Normal University, Guangzhou, China; ^3^Key Laboratory of Brain, Cognition and Education Sciences (South China Normal University), Ministry of Education, Guangzhou, China; ^4^Guangdong Key Laboratory of Mental Health and Cognitive Science, South China Normal University, Guangzhou, China

**Keywords:** neural pattern similarity, native language, word learning, fusiform gyrus, fMRI

## Abstract

**Introduction:**

Previous neuroimaging studies on bilingualism revealed that individuals tend to apply their native-language (L1) neural strategies to second language (L2) learning and processing. Nevertheless, it is still unclear how the utilization of the L1 neural strategies affects visual word learning in a new language.

**Methods:**

To address this question, the present study scanned native Chinese speakers while performing implicit reading tasks before 9-day form-meaning learning in Experiment 1 and before 12-day comprehensive word learning in Experiment 2. To quantify the application of the L1 neural strategies in novel word learning, representational similarity analysis (RSA) was used to compute the neural pattern similarity (PS) between the L1 and artificial language (i.e., cross-language PS) before training.

**Results:**

Univariate analysis revealed that reading both Chinese words (CWs) and artificial language words (ALWs) elicited activations in a typical reading network. More importantly, RSA revealed that greater pre-training cross-language PS in the left fusiform gyrus was associated with higher learning rate.

**Discussion:**

These findings directly reveal the facilitating role of the L1 neural strategies in novel word learning and further extend the assimilation hypothesis from the utilization of the L1 neural network in L2 learning to its learning outcomes.

## 1 Introduction

Learning to read novel words is critical for the acquisition of written language. Individuals behaviorally show significant individual differences when they learn to read novel words. Numerous neuroimaging studies have identified neural underpinnings for such individual differences. They revealed significant correlations between individuals’ learning performance and neural activity or anatomical structure of certain brain regions involved in word reading, including the left postcentral gyrus, inferior frontal gyrus, superior temporal gyrus, middle temporal gyrus, and fusiform gyrus (Asaridou et al., 2016; Chai et al., 2016; Chyl et al., 2018; Deng et al., 2016; Ekerdt et al., 2020; Hofstetter et al., 2017; Li et al., 2021; Martin et al., 2019; Palomar-García et al., 2017). For example, Martin et al. (2019) constructed a second writing system that is perceptually atypical by using house images as letters. They performed nine sessions to train English speakers in reading HouseFont and asked them to conduct a passive viewing task before and after the training sessions. Results showed that changes in activation within the visual word form area (VWFA) from pre-training to post-training predicted the reading speed of HouseFont after training. In addition to neural changes induced by learning, neural activation patterns before learning have been found to be predictive of novel word learning (Barbeau et al., 2017; Dong et al., 2008; Mei et al., 2008; Qu et al., 2017). For example, in the study conducted by Xue et al. (2006), Chinese speakers were trained to learn the visual forms of an artificial language that was created using Korean Hangul characters for 2 weeks. They found that pre-training fusiform asymmetry predicted behavioral performance after training. Similarly, Barbeau et al. (2017) revealed that the pre-training neural activations in the left inferior parietal lobule predicted behavioral improvement in French reading in native English speakers after 12-week training. These findings of pre-training neurofunctional predictors seem to indicate that pre-existing neural strategies have an impact on subsequent learning.

Although much effort has been devoted to neurofunctional predictors of individual differences in novel word learning (Chen et al., 2007; Palomar-García et al., 2017; Qi et al., 2019; Rodriguez et al., 2018; Xue et al., 2006), it is still unclear how the L1 neural strategies contribute to those for novel word learning. Prior research on bilingualism has highlighted the critical role of the existing L1 experience in shaping brain activation when reading L2 words (Mei et al., 2015; Nakada et al., 2001; Tan et al., 2003). To elucidate the effects of the L1 on L2 learning, the assimilation hypothesis proposed by Perfetti et al. (2007) assumed that individuals tended to apply the neural network for L1 (i.e., the L1 network) to L2 learning (i.e., the assimilation process). Consistent with this view, studies found that, similar activation patterns to L1 (i.e., Chinese) were elicited in the left middle frontal gyrus (a region presumably for phonology processing of Chinese characters) and bilateral fusiform gyrus for Chinese-English bilinguals when reading L2 words (i.e., English) (Cao et al., 2013; Nelson et al., 2009; Tan et al., 2003). Numerous studies have further shown that greater involvement in the L1 network was found to be associated with higher proficiency in L2 (Cao et al., 2013; Gao et al., 2017; Kim et al., 2017; Qu et al., 2019; Stein et al., 2009). These findings demonstrated that individuals utilized their L1 neural network when reading L2 words.

Despite accumulating evidence for applying the L1 network to L2 word reading, it still remains unclear whether the use of the L1 network facilitates novel word learning. To address this question, the current study used cross-language pattern similarity (PS) as an index to quantify the degree of the L1 network applied in learning to read words in a new language. Representational similarity analysis (RSA) is a multivariate analysis method that allows for the examination of finer-grained activity patterns across multiple voxels compared to traditional univariate activation analysis (Kriegeskorte, 2008; Kriegeskorte and Kievit, 2013). This method has been widely used to investigate neural representations across different conditions or stimuli, particularly in studies of perception, memory, and language processing (Gauthier and Tarr, 2016; Kriegeskorte and Kievit, 2013; Li et al., 2022; Yang et al., 2024). The cross-language PS was computed by using RSA to assess the similarity in multi-voxel activation patterns across different languages. By quantifying cross-language PS, previous studies have demonstrated that factors such as orthographic transparency (Dong et al., 2021), language proficiency (Li et al., 2019; Qu et al., 2019), and the depth of semantic processing (Li et al., 2023) modulated the cross-language PS between native and non-native languages in word reading. In the present study, the greater cross-language PS in a certain brain region indicates that more neural strategies of L1 are applied to word learning in a new language. Thus, the question of whether the L1 network affects the learning efficiency of novel words could be addressed by examining the relation between cross-language PS and individuals’ learning performance.

Consequently, utilizing fMRI and RSA, the present study aimed to investigate how L1 neural strategies contribute to novel word learning. Two experiments were conducted in which an artificial language training paradigm was adopted. In Experiment 1, native Chinese speakers were asked to conduct 9-day training sessions in which they had to learn the form-meaning associations between visual forms and semantics of artificial language words (ALWs), and the learning performance in each day was assessed by using a semantic decision task. Imaging data were collected by using an implicit reading (i.e., color judgment) task before training. Due to the crucial role in word reading (Cohen et al., 2002; Cohen and Dehaene, 2004; Dehaene and Cohen, 2011) and visual word learning (Qu et al., 2017, 2019; Xue et al., 2006), the bilateral fusiform gyrus were selected as regions of interest (ROIs) in this study. The role of the L1 neural strategies in novel word learning was then examined by correlating pre-training neural PS between Chinese words (CWs) and ALWs in the bilateral fusiform gyrus with participants’ learning performance. Experiment 2 aimed to extend the findings in Experiment 1 from form-meaning learning to more comprehensive learning (i.e., learning the visual forms, phonologies, and semantics of novel words together). In Experiment 2, we conducted a re-analysis of the data from Qu et al. (2017), in which participants learned the visual forms, phonologies, and semantics of 30 ALWs for 12 days. As in Experiment 1, we performed correlation analysis to investigate the contributions of the L1 neural strategies in novel word learning.

## 2 Experiment 1

Experiment 1 trained native Chinese speakers to learn form-meaning associations of ALWs to examine whether neural PS between the L1 (i.e., Chinese) and artificial languages correlated with their learning performance. Based on the assimilation hypothesis (Perfetti et al., 2007) and previous findings of the established role of the left fusiform gyrus in visual word learning (Qu et al., 2017, 2019; Xue et al., 2006), we expected that cross-language PS before training could predict individuals’ learning outcomes.

### 2.1 Materials and methods

#### 2.1.1 Participants

Twenty-six native Chinese college students (15 females, 21.88 ± 2.2 years old) participated in this experiment, all of whom had learned English as their L2. They were asked to rate their proficiency in both languages on a 7-point scale (1 = “quite poor,” 7 = “highly proficient”). The average ratings were 6.49 (SD = 0.83) for Chinese and 3.66 (SD = 0.76) for English. Thus, the participants were unbalanced Chinese-English bilinguals with intermediate proficiency in English. None of them had any prior experience in Korean. All participants were right-handed as assessed by the Edinburgh handedness test (Snyder and Harris, 1993), had normal or corrected-to-normal vision, and had no history of psychiatric disorders. Prior to participation in the experiment, a signed and dated written informed consent was obtained from all participants. In addition, this experiment was approved by the IRB of the School of Psychology at South China Normal University.

#### 2.1.2 Materials

Two types of materials were selected for this experiment, including 20 CWs and 60 ALWs. All CWs were medium-to high-frequency single characters, with an average of 37.80 per million (SD = 77.08). Moreover, these characters consisted of 7–13 strokes, with an average of 9.65 (SD = 1.72). The ALWs were created by borrowing visual forms of 60 single characters of Korean Hangul, which composed of 22 Hangul letters and consisted of 6–10 strokes, with an average of 8.20 (SD = 0.96). The ALWs were composed of 12 consonants and 10 vowels. In order to minimize the potential confounding effects of learning materials, we divided 60 ALWs into three matched sets (each had 20 words) and randomly assigned them to participants for learning. Each participant learned one set of materials. The three sets were strictly matched in terms of visual complexity (i.e., number of strokes) [*F*_(2,59)_ = 1.20, *p* = 0.310]. Twenty pictures from four categories (i.e., plants, animals, instruments, and stationery) were selected and assigned to the Korean Hangul characters to construct form-meaning associations for training. The assignment of three training sets was counterbalanced across participants.

#### 2.1.3 Training procedure

Participants were instructed to learn the associations between visual forms (i.e., Korean Hangul characters) and semantics of the ALWs over 9 days, with a series of learning tasks lasting about 1 h each day. Using a computerized program, a series of tasks were conducted to help participants to learn form-meaning associations, including handwriting (writing the ALWs carefully), character learning (learning the ALWs and their meanings), free learning (learning the ALWs with which participants had difficulties), semantic choice (selecting the accurate meaning from four options to match the target character), form judgment (judging whether the two sequentially presented words was same or different), form-meaning recall (recalling the meaning of the word presented on the screen and then judging whether it is correct based on feedback), and fast matching (matching characters with their corresponding meanings as accurately and quickly as possible).

#### 2.1.4 Behavioral task

To assess participants’ learning performance during training, they were asked to complete a semantic decision task at the end of each day. Participants had to categorize ALWs by pressing one of the four keys (i.e., “1” for plants, “2” for stationary, “3” for animals, and “4” for instruments). The reaction time and accuracy were recorded during the task.

Considering that the reaction time might reflect general naming speed rather than novel word learning, we used the rate of learning as an index to evaluate participants’ learning performance. For each participant, a learning curve was used to fit his/her reaction time over 9 learning sessions to quantify the learning rate. Specifically, the non-linear learning curve was modeled using a power function (y = a^∗^x^−b^), with ‘a’ and ‘b’ representing the initial learning performance and the learning rate, respectively (Anderson, 1983; Logan, 1988). The coefficient of determination was used to determine goodness-of-fit.

#### 2.1.5 fMRI task

Participants were asked to perform an implicit reading (i.e., color judgment) task in the scanner before training. This task involved two types of stimuli (i.e., ALWs and CWs). As mentioned in “Materials,” each participant was assigned one of three sets of ALWs. Each set contained 20 items and was presented three times by conducting three runs. Similarly, CWs were scanned three times in three repeated runs. Participants were instructed to perform six runs in total. Matlab (Mathworks) was used to present the stimulus and collect the response data.

**Figure 1:**
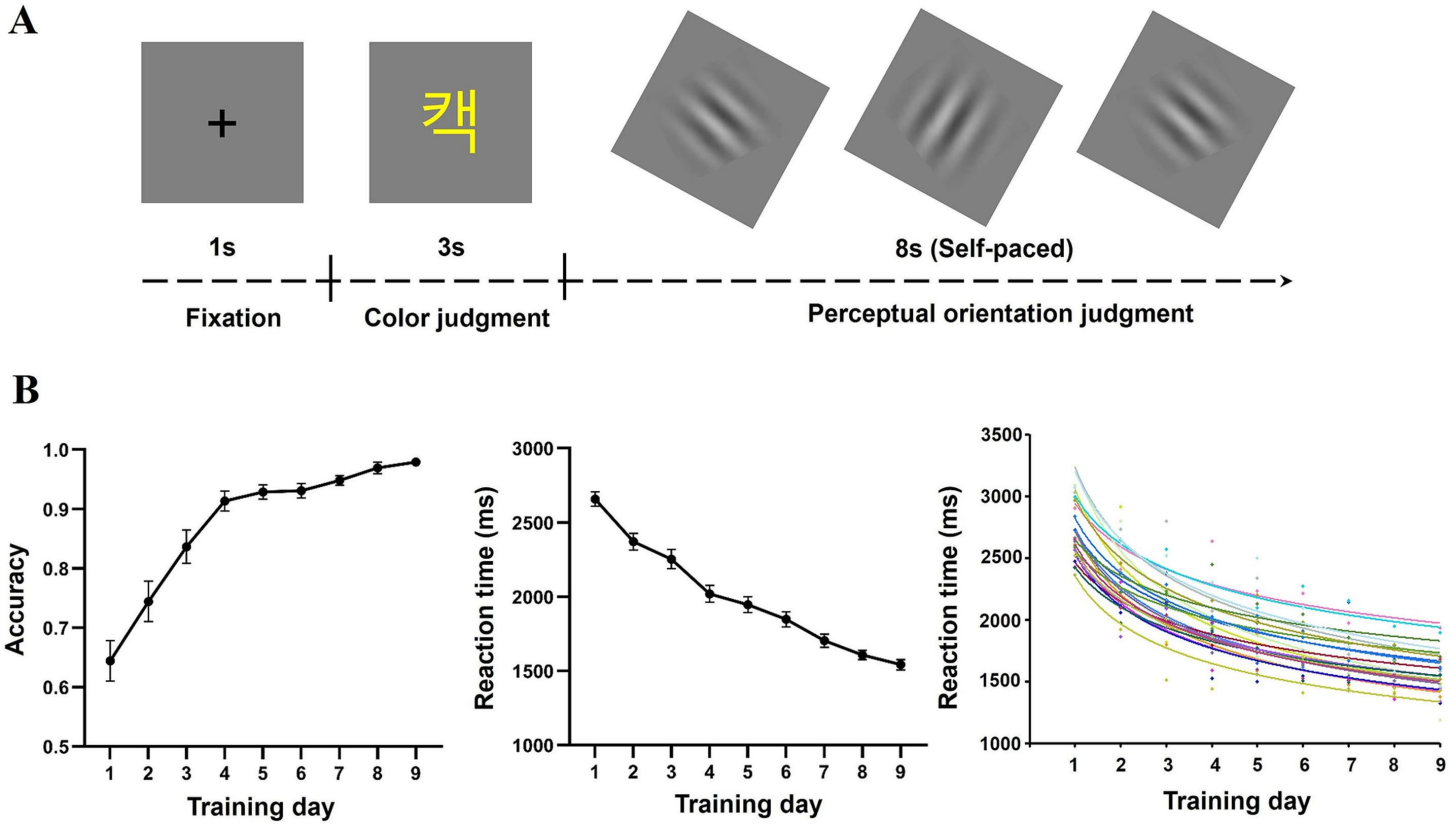
The task during the fMRI scan **(A)** and learning performance at each training day **(B)**. The bottom panels display reaction time, accuracy, and the fitted learning curve for each participant in the semantic decision task.

There were, in total, six functional runs, with three runs for CWs and three runs for ALWs. Twenty words in one language were presented in each run and repeated three times across runs. In this experiment, a slow event-related design was adopted (see Figure 1A). Each trial began with a fixation for 1 s, followed by the stimulus for 3 s. Participants were asked to judge whether the color of the stimulus was white or yellow by pressing the two corresponding buttons (i.e., “1” for yellow, “4” for white). The correspondence between color and button was counterbalanced across participants. After that, participants were instructed to complete an 8 s self-paced perceptual orientation judgment task, which was used to construct a slow event-related design and avoid further rehearsing the presented stimulus (Dong et al., 2020; Li et al., 2022; Liu et al., 2021; Qu et al., 2021). This task asked participants to determine the orientation of the presented Gabor image, which was randomly tilted either 45° to the left or the right. After participants responded, the Gabor image disappeared, and then the subsequent image was presented on the screen. Each trial lasted for 12 s, and the whole experiment lasted for 24 min in total.

#### 2.1.6 MRI data acquisition

A 3 T Siemens scanner was used to collect imaging data at the MRI Center of South China Normal University. Functional images were obtained by using a T2*-weighted single-shot EPI sequence, with the following parameters: flip angle = 90°, TR = 2000 ms, TE = 25 ms, FOV = 192 × 192 mm, image matrix = 64 × 64, slice thickness = 3.5 mm, slice number = 35. The Anatomical MRI was acquired using a T1-Weighted, three-dimensional, gradient-echo pulse sequence with specific parameters: flip angle = 9°, TR = 2,300 ms, TE = 3.24 ms, FOV = 256 × 256 mm, image matrix = 256 × 256, slice thickness = 1 mm, slice number = 176.

#### 2.1.7 Image preprocessing and statistical analysis

FEAT (FMRI Expert Analysis Tool) Version 6.00, a part of FSL (FMRIB’s Software Library^1^), was used for the preprocessing of the MRI data. The first functional volume of each run was automatically excluded to allow for T1 equilibration. Then the remaining data from each participant were realigned to reduce the confounding effects of small head movements. All images were spatial smoothed with a Gaussian kernel of 5-mm full-width-half-maximum (FWHM) and were then temporally filtered using a 100 s non-linear high-pass filter. Next, two registration steps were conducted, including registering functional images to each participant’s structural images and then standardizing these images into the Montreal Neurological Institute (MNI) template (Jenkinson and Smith, 2001).

Group activations of CWs and ALWs were generated by using a three-level analysis. At the first level, a general linear model (GLM) was used to estimate the preprocessed data in each run for each participant. By convolving the onsets and durations of events with double-gamma hemodynamic response function (HRF), regressors were generated as predictors in the GLM. To improve statistical sensitivity, six head motion parameters (i.e., 3 rotations and 3 translations) and temporal derivatives were employed as covariates of no interest.

At the second level, the fixed-effects model was constructed to obtain the mean activations across three runs of each condition (i.e., CWs or ALWs) and to calculate two contrasts (i.e., CWs minus ALWs and ALWs minus CWs) to explore the specific neural activations for each type of material. Finally, group activations were estimated across participants using a random-effects model. All activations were thresholded with a height threshold of z > 2.6 and a cluster probability, *p* < 0.05, corrected for whole-brain multiple comparisons using the Gaussian random field (GRF) theory (Worsley, 2001).

#### 2.1.8 Region-of-interest-based representational similarity analysis

To investigate whether neural PS between native and artificial languages predicted learning performance, we performed region-of-interest-based (ROI-based) RSA. In this analysis, cross-language PS was calculated to quantify the degree of similarity in neural activations between CWs and ALWs during word reading. As discussed in Introduction, because of the critical role of the bilateral fusiform cortex in word reading and visual word learning (Cohen et al., 2002; Cohen and Dehaene, 2004; Dehaene and Cohen, 2011; Qu et al., 2017, 2019; Xue et al., 2006), they were anatomically defined as regions of interest (ROIs) based on the Harvard–Oxford probabilistic atlas (Maximal Probability Threshold: 25%) within FSL.

In RSA, the first-level models in the above univariate activation analysis were re-estimated with unsmoothed data. Each item in each run was modeled as a single regressor to precisely estimate the hemodynamic response mode (HRF). The contrast of parameter estimates (COPE) values of each item were then extracted from each voxel in the bilateral fusiform gyrus for each run. These values were averaged across 3 runs for each condition (i.e., CWs and ALWs) to obtain neural activity of single trials with relatively higher signal-to-noise ratio. Cross-language PS was computed by correlating (Pearson correlation) the COPE values of every pair of cross-language word pairs and then transformed into Fisher’s Z-scores. Finally, Pearson correlation analysis was performed on the pre-training cross-language PS and learning performance (i.e., the rate of learning).

### 2.2 Results

#### 2.2.1 Training improved behavioral performance during reading

One-way repeated ANOVA was conducted on reaction times and accuracy in the semantic decision task. As expected, training significantly reduced reaction time [*F*_(8,200)_ = 93.66, *p* < 0.001, *η^2^_p_* = 0.789] and improved accuracy [*F*_(8,200)_ = 40.94, *p* < 0.001, *η^2^_p_* = 0.621], suggesting that the training was effective (Figure 1B). It is worth noting that the accuracy was higher than 0.9 after 9 days training, suggesting that participants acquired the form-meaning associations of ALWs.

To assess the learning rate, we fitted the learning curve to each participant’s reaction times (Figure 1B). Data from four participants were excluded because their goodness-of-fit was lower than 0.7. Thus, data from 22 participants were used in subsequent analysis. The mean goodness-of-fit for the remaining participants was 0.84.

#### 2.2.2 Neural activations for CWs and ALWs during reading

**Figure 2:**
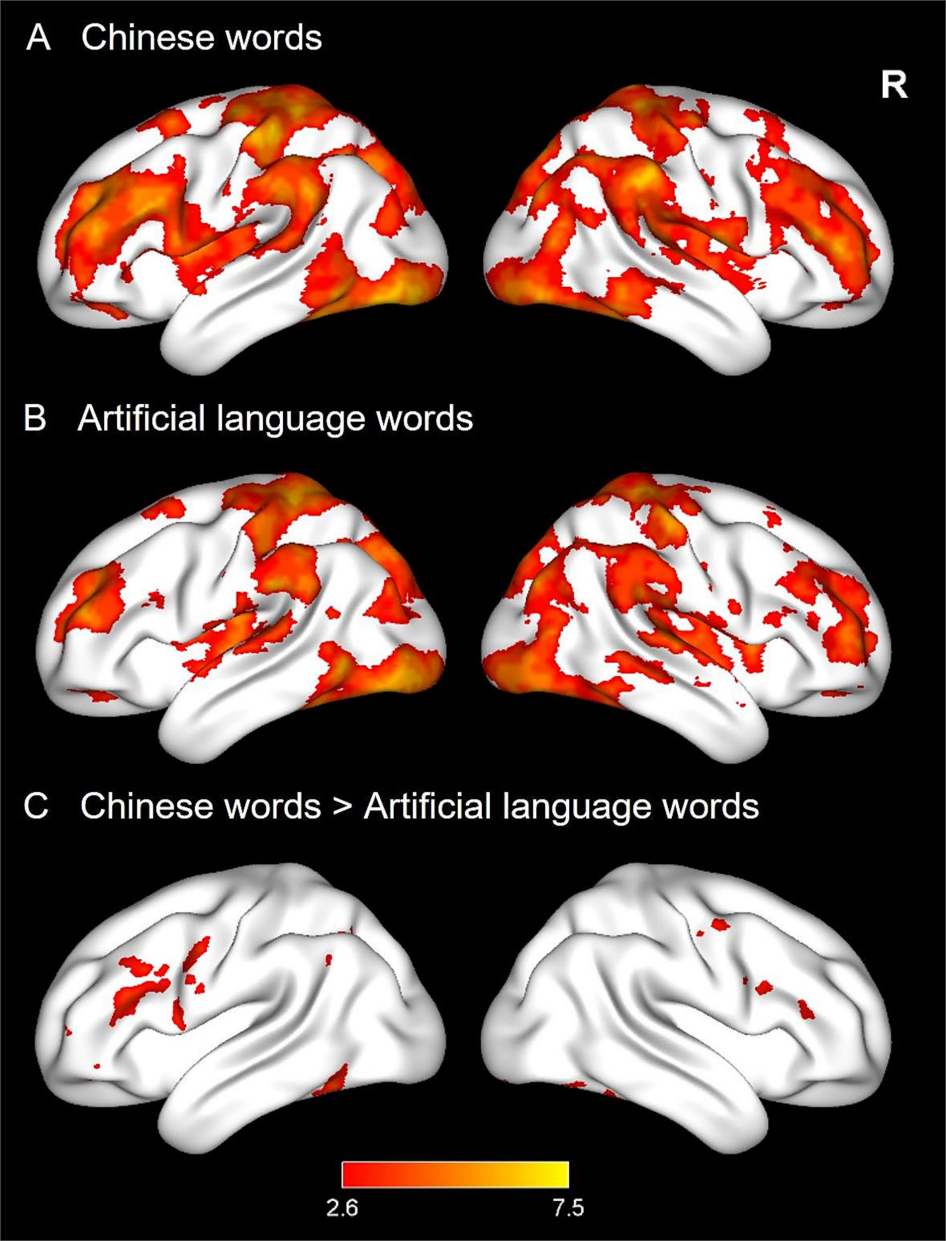
Brain activations of word reading in Experiment 1. Three panels display neural activations of Chinese words (CWs, **A**), and artificial language words (ALWs, **B**), as well as stronger activations for CWs relative to ALWs **(C)**.

Whole-brain activation analysis was conducted to explore neural activations for CWs and ALWs. We found that reading both CWs and ALWs elicited extensive activations in the typical reading network (see Figures 2A,B), including the bilateral prefrontal cortex, occipitotemporal cortex, and temporoparietal cortex. Direct comparison across the two languages revealed that CWs elicited greater activation than ALWs in the bilateral prefrontal cortex, fusiform gyrus, and left inferior temporal gyrus. In contrast, no regions were observed in the reverse contrast (see Figure 2C).

#### 2.2.3 Cross-language PS before training predicted the rate of learning

**Figure 3:**
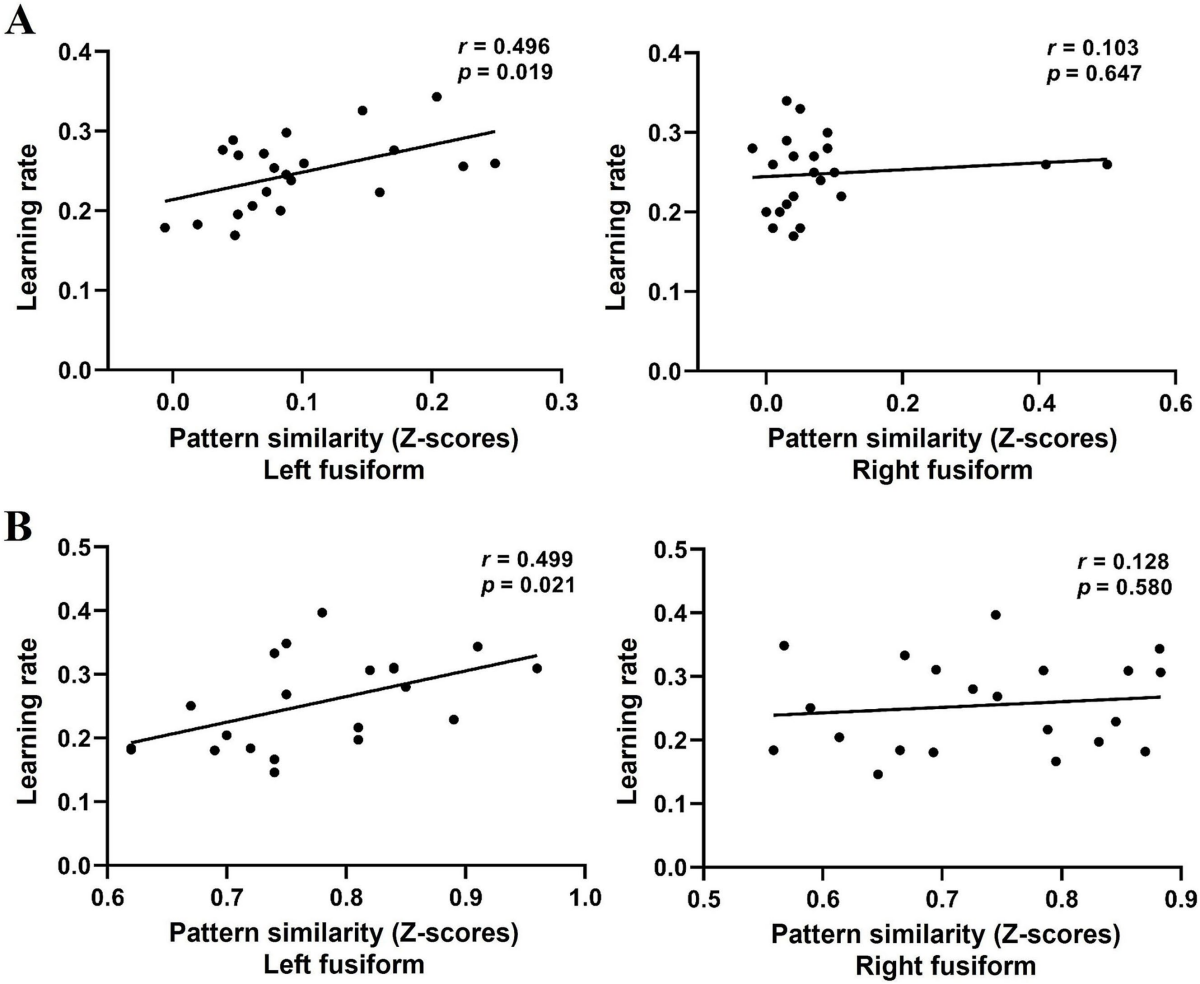
Scatter plots of the learning rate and cross-language PS in the left and right fusiform gyrus in Experiments 1 **(A)** and 2 **(B)**.

Finally, we examined whether the application of L1 network facilitates novel word learning by correlating cross-language PS in the bilateral fusiform gyrus before training with learning performance (i.e., the rate of learning). Results showed that cross-language PS in the left fusiform gyrus (*r* = 0.496, *p* = 0.019), but not in the right fusiform gyrus (*r* = 0.103, *p* = 0.647; Figure 3A), was positively correlated with the learning rate. These results indicate that greater neural PS to the L1 during initial learning leads to faster learning of novel words. We also selected several additional language-related regions based on the Harvard-Oxford probabilistic atlas as ROIs for analysis, including the left pars opercularis, pars triangularis, angular gyrus, middle temporal gyrus, and inferior temporal gyrus, but found no significant correlations (*ps* > 0.05).

### 2.3 Discussion

Using the form-meaning artificial language training paradigm, Experiment 1 examined whether the use of the L1 neural strategies affected novel word learning. The univariate activation analysis revealed that, consistent with prior findings (Mei et al., 2014; Mei et al., 2015), reading both CWs and ALWs elicited similar activations in a typical word reading network, suggesting the potential occurrence of the assimilation process (Perfetti et al., 2007). More importantly, as expected, we found that cross-language PS in the left fusiform gyrus, but not in its right homologue, was positively associated with the rate of learning. These results confirmed the important role of the left fusiform gyrus in visual word learning (Qu et al., 2017, 2019; Xue et al., 2006) and further indicated that the application of the L1 neural strategies facilitates novel word learning.

It should be noted that there were at least two limitations in Experiment 1. First, ALWs lacking phonologies would restrict the generalization of our findings to natural word learning to some degree. Second, the learning performance was collected by using a semantic decision task in Experiment 1. Assessing the learning performance with a single reading task might involve task-specific processes. To overcome those two limitations, Experiment 2 re-analyzed the data in Qu et al. (2017), in which participants had to learn visual forms, phonologies, and semantics of ALWs, and individuals’ learning performance was assessed using two tasks, to further validate the predictive role of cross-language PS in learning to read novel words.

## 3 Experiment 2

In Experiment 1, we found L1 neural strategies could facilitate individuals’ learning performance. Experiment 2 aimed to further validate those findings by using a more comprehensive learning paradigm and assessing the learning performance with two phonological (i.e., word naming and picture naming) tasks. In this experiment, we re-analyzed the data from Qu et al. (2017), in which participants were required to learn the visual forms, phonologies, and semantics of ALWs.

### 3.1 Materials and methods

#### 3.1.1 Participants

In Experiment 2, 24 native Chinese college students (13 females) who had acquired English as their L2 were recruited, with a mean age of 19.46 years (SD = 0.93) and a range of 18 to 22. As in Experiment 1, all participants rated their proficiency in two languages using a 7-point scale. The average proficiency ratings were 5.5 (SD = 1.06) for Chinese and 3.47 (SD = 0.69) for English, indicating that the participants were unbalanced Chinese-English bilinguals and had an intermediate proficiency in English. All participants had no prior experience with Korean and were strongly right-handed with normal or corrected-to-normal vision. All of them had no previous history of psychiatric disease and provided written informed consent before participating in this experiment. This experiment received approval from the IRB at the School of Psychology, South China Normal University.

#### 3.1.2 Materials and training procedure

Three types of words were used in this experiment, including 30 ALWs, 30 CWs, and 30 English words (EWs). EWs were included for other purposes and consequently excluded in the subsequent analysis. CWs were single character words with medium-to high-frequency and consisted of 6–9 strokes. ALWs were constructed by borrowing the visual forms and phonologies of 30 Korean Hangul characters which consisted of 5–9 strokes and 2–3 units. They were strictly matched with Chinese characters in terms of number of strokes and number of units. The original correspondence between visual forms and phonologies was shuffled. In addition, 30 pictures of objects served as meanings and were randomly assigned to the ALWs.

Participants were instructed to complete a comprehensive training in which they had to learn visual forms, phonologies, and semantics of 30 ALWs for about an hour each day for a total of 12 days. A number of learning tasks were conducted to improve participants’ learning efficiency. Please refer to Qu et al. (2017) for more details about materials and training procedures.

#### 3.1.3 Behavioral tasks

To assess participants’ learning performance, two phonological (i.e., word naming task and picture naming) tasks were performed at the end of each training day. Two tasks were used to assess the learning performance to reduce the potential confounding effects of task-specific processes. In the word naming task, participants had to name 30 ALWs in Korean, while in the picture naming task, they had to name 30 pictures in Korean. For both tasks, the reaction time (RT) and accuracy were recorded. As in Experiment 1, the learning rate was fitted to quantify individuals’ learning performance. To improve representativeness and consistency of results, the mean RTs of two naming tasks were calculated as naming speed to further fit the learning curve by using the power function (y = a·x^-b^).

#### 3.1.4 fMRI task

An implicit reading (i.e., a passive viewing) task was performed during an fMRI scan before training. The task involved 30 CWs, 30 EWs, and 30 ALWs. In addition, nine fillers (i.e., underlined words) were also included for key pressing to keep participants attentive to the task. This experiment adopted an event-related design and optimized trial sequence with OPTSEQ2^2^ (Dale, 1999).

During scanning, each stimulus was displayed for 600 ms. Following the stimulus, a fixation was presented on the screen, lasting for 1–5 s (mean = 2 s). Participants had to watch the stimuli and press the response button once the word was underlined. Each stimulus was presented twice. In total, 189 stimuli were presented, lasting 566 s.

#### 3.1.5 MRI data acquisition and analysis

Imaging data were acquired using a 3.0 T Siemens magnetic resonance scanner at the MRI Center of South China Normal University. The functional and structural imaging acquisition sequence and parameters were the same as in Experiment 1. A total of 208 slices were obtained to provide a high-resolution structural image of the whole brain.

Functional images were analyzed with FEAT (FMRI Expert Analysis Tool) 6.0 of FSL (FMRIB’s Software Library^3^). The first three volumes of the scan were automatically deleted for T1 equilibrium effects, and then the remaining images were as in Experiment 1.

The preprocessed data were then estimated by using a general linear model, in which three regressors (i.e., CWs, EWs, and ALWs) were included. To avoid potential confounding effects, the underlined words (i.e., fillers) were modeled as nuisance variables. Group activations were obtained by using the same procedure as in Experiment 1.

As in Experiment 1, representational similarity analysis was also conducted within the bilateral fusiform gyrus. All RSA procedures remained consistent with those in Experiment 1 except that cross-language PS was obtained by computing the neural PS between the averaged activation patterns of CWs and that of ALWs.

### 3.2 Results

#### 3.2.1 Training improved behavioral performance during reading

Behavioral data had been reported in Qu et al. (2017). Similar to Experiment 1, results showed that artificial language training significantly reduced reaction times and improved the accuracy of ALWs in both naming tasks. In addition, we fitted the learning curve to each participant’s mean RTs for the two naming tasks. Three participants’ data were removed from the subsequent correlation analysis as their goodness-of-fit was lower than 0.7.

#### 3.2.2 Neural activations for CWs and ALWs before training

**Figure 4:**
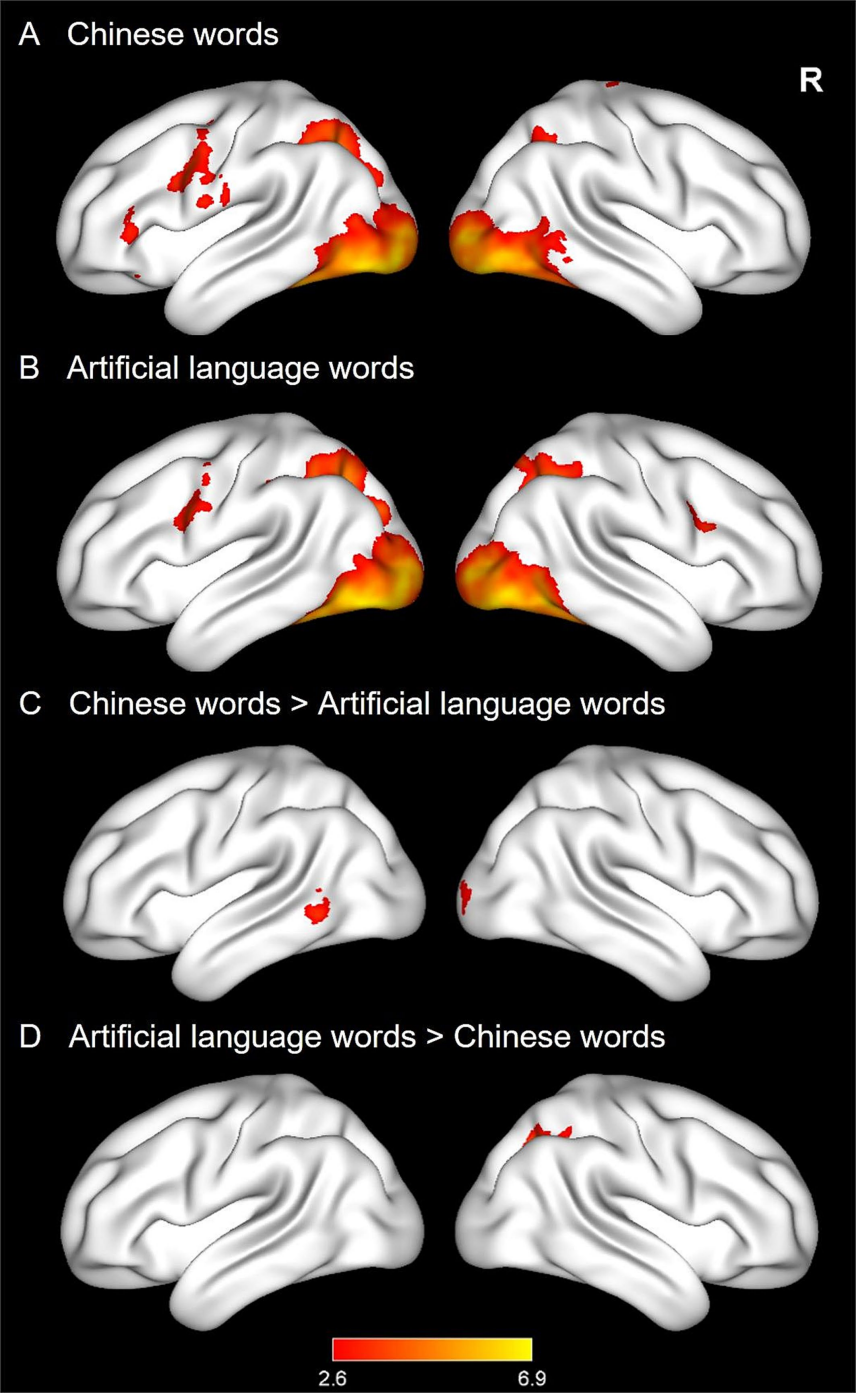
Whole-brain activation maps in Experiment 2. The four panels show activations for Chinese words (CWs, **A**), and artificial language words (ALWs, **B**), stronger activations for CWs **(C)**, as well as stronger activations for ALWs **(D)**.

As in Experiment 1, reading CWs and ALWs generally elicited a similar reading network, including the left prefrontal cortex, occipitoparietal cortex, and occipitotemporal cortex (see Figures 4A,B). Further comparisons across the two conditions revealed that CWs showed greater activations in the left middle temporal gyrus (MTG) and right occipital cortex than ALWs (see Figure 4C). In contrast, ALWs elicited greater activations in the right superior occipital cortex (see Figure 4D).

#### 3.2.3 Cross-language PS before training predicted the rate of learning

Finally, we performed Pearson correlation analysis to further investigate whether cross-language PS before training was predictive of the learning rate in the naming tasks. Results confirmed our findings in Experiment 1. Specifically, cross-language PS in the left fusiform gyrus before training was found to be positively correlated with the rate of learning (*r* = 0.499, *p* = 0.021), but it was not significant in the right fusiform gyrus (*r* = 0.128, *p* = 0.580; Figure 3B). As in Experiment 1, additional language-related regions as ROIs were also selected for analysis but found no significant correlations (*ps* > 0.05).

### 3.3 Discussion

Experiment 2 aimed to verify the predictive role of cross-language PS by using a comprehensive artificial language training and assessing the learning performance with two naming tasks. First, we found that, similar to the results in Experiment 1, reading both CWs and ALWs elicited activations in the bilateral prefrontal cortex, superior parietal cortex, and occipitotemporal cortex. It should be noted that, relative to Experiment 1, the activations in Experiment 2 showed a bit narrow distribution, which could be attributed to the decreased cognitive loads in the passive viewing task relative to the color judgment task.

More importantly, Experiment 2 replicated the predictive role of cross-language PS in novel words found in Experiment 1, suggesting that the facilitating role of the L1 neural strategies was not specific to form-meaning learning or the particular behavioral assessment. Overall, the findings from both experiments suggest that the application of the L1 neural strategies during initial learning facilitates novel word learning.

## 4 General discussion

By combining an artificial language training paradigm and RSA, the current study investigated how the use of L1 neural strategies affected learning to read novel words. In both experiments, reading CWs and ALWs elicited activations in the bilateral prefrontal cortex, occipitoparietal cortex, and occipitotemporal cortex, although there were subtle differences in spatial distribution across the two experiments. More importantly, multivariate RSA revealed that pre-training cross-language PS in the left fusiform gyrus, but not in the right fusiform gyrus, was positively correlated with individuals’ learning outcomes (i.e., the rate of learning). These convergent findings suggest that the utilization of the L1 neural strategies facilitates the acquisition of novel words.

The results of our study made several significant contributions. First, the current study confirmed the critical involvement of the left fusiform gyrus in the process of visual word learning from the perspective of the impacts of L1 on word learning in a new language (Baeck et al., 2015; Cohen et al., 2002; Dehaene et al., 2010; Hannagan et al., 2015; Li et al., 2021; Mei et al., 2015; Murphy et al., 2019; Qu et al., 2017, 2019). Much research on reading in normal children, adults, and brain lesion patients has suggested the crucial involvement of the left fusiform gyrus in visual form processing (Bai et al., 2011; Dehaene et al., 2001; Gaillard et al., 2006; Ma et al., 2011; Mani et al., 2008; Pflugshaupt et al., 2009; Richlan et al., 2011; Vigneau et al., 2005). Researchers even labeled the mid-fusiform gyrus as visual word form area (VWFA) and posited that it is specifically responsible for the processing of visual forms (Cohen et al., 2002; Dehaene and Cohen, 2011). Further research on word memory and word learning has established the associations between the activations in the left fusiform gyrus and individuals’ behavioral performance of learning and memory (Li et al., 2021; Mei et al., 2010; Qu et al., 2017, 2019; Xue et al., 2006). Specifically, it has been found that greater activation in that brain region was associated with better performance in learning and memory (Qu et al., 2017, 2019). Here, we used multivariate RSA to confirm the left fusiform gyrus’s predictive role of the neural activation pattern in novel visual word learning from the perspective of the impact of L1 on new language learning. In contrast, the right fusiform gyrus, a region that has been repeatedly found to be responsible for visuospatial processing (Dehaene et al., 2001, 2004; Mei et al., 2015; Nelson et al., 2009), did not show a significant correlation.

More importantly, by quantifying cross-language PS before learning using RSA, the study, for the first time, demonstrated that the use of L1 neural strategies has a positive impact on novel word learning. By calculating the neural PS between the native and new languages during initial learning, cross-language PS measured the degree of the L1 neural strategies applied to new language learning. In this study, we consistently observed positive correlations between pre-training cross-language PS in the left fusiform gyrus and novel word learning in two experiments. These convergent results suggest that the neural computations in the left fusiform gyrus tuned years of reading experience in the L1 are optimal for visual word learning in a new language. It provides direct neuroimaging evidence for the positive influence of L1 neural strategies on novel word learning.

Finally, our results extended the assimilation hypothesis from the utilization of L1 networks in L2 learning to its outcomes on learning efficiency. As mentioned in Introduction, prior studies on bilingualism revealed that bilinguals tend to apply the L1 neural network to L2 learning, known as the assimilation process (Nelson et al., 2009; Perfetti et al., 2007). Further studies have suggested that proficiency in L2 was positively associated with the involvement of the L1 network in L2 processing (Cao et al., 2013; Gao et al., 2017; Kim et al., 2017; Li et al., 2021; Qu et al., 2019). These studies provided evidence for the occurrence of the assimilation processing during L2 learning. Here, utilizing an artificial language paradigm and RSA, the current study demonstrated that the degree of assimilation has an important impact on the learning efficiency of a new vocabulary. Specifically, the more similar the activity patterns of learners’ L1 and L2 (i.e., greater cross-language PS) in the left fusiform cortex, the better they achieved in novel word learning. These findings not only corroborated but also extended the assimilation hypothesis.

Two potential limitations of our study should be discussed. First, the materials of ALWs were created by the visual forms of Korean characters, which, to some extent, were visually similar to participants’ L1 (i.e., Chinese). It remains unclear whether our findings could be generalized to other writing systems (e.g., English and Spanish) which are different from Chinese in orthography. Thus, future research should replicate our findings in writing systems with different orthographies. Second, participants were asked to complete a relatively short-term training (i.e., 9 and 12 days) in this study. It is not clear whether the application of the L1 neural strategies affects long-term learning outcomes. Future research should address this question by following up on word learning for a relatively longer period.

In conclusion, the present study revealed that greater PS to the L1 before training was associated with better learning outcomes. These findings extend the assimilation hypothesis and suggest that the use of the L1 neural strategies during initial learning facilitates subsequent novel word learning.

## Data Availability

The raw data supporting the conclusions of this article will be made available by the authors, without undue reservation. The data that support the findings of this study are available at: https://osf.io/dksfe/.

## Footnotes

^1^ www.fmrib.ox.ac.uk/fsl

^2^ http://surfer.nmr.mgh.harvard.edu/optseq2/

^3^ http://www.fmrib.ox.ac.uk/fsl
